# Socioeconomic inequality in short birth interval in Ethiopia: a decomposition analysis

**DOI:** 10.1186/s12889-020-09537-0

**Published:** 2020-10-06

**Authors:** Desalegn Markos Shifti, Catherine Chojenta, Elizabeth G. Holliday, Deborah Loxton

**Affiliations:** 1 Saint Paul’s Hospital Millennium Medical College, Addis Ababa, Ethiopia; 2 Priority Research Centre for Generational Health and Ageing, School of Medicine and Public Health, University of Newcastle, Newcastle, NSW Australia; 3 Centre for Clinical Epidemiology and Biostatistics, School of Medicine and Public Health, University of Newcastle, Newcastle, NSW Australia

**Keywords:** Socioeconomic inequality, Concentration index, Concentration curve, Decomposition analysis, Short birth interval, Ethiopia

## Abstract

**Background:**

Short birth interval, defined as a birth-to-birth interval less than 33 months, is associated with adverse maternal and child outcomes. Evidence regarding the association of maternal socioeconomic status and short birth interval is inconclusive. Factors contributing to the socioeconomic inequality of short birth interval have also not been investigated. The current study assessed socioeconomic inequality in short birth interval and its contributing factors in Ethiopia.

**Methods:**

Data from 8448 women collected in the 2016 Ethiopia Demographic and Health survey were included in the study. Socioeconomic inequality in short birth interval was the outcome variable. Erreygers normalized concentration index (ECI) and concentration curves were used to measure and illustrate socioeconomic-related inequality in short birth interval, respectively. Decomposition analysis was performed to identify factors explaining the socioeconomic-related inequality in short birth interval.

**Results:**

The Erreygers normalized concentration index for short birth interval was − 0.0478 (SE = 0.0062) and differed significantly from zero (*P* < 0.0001); indicating that short birth interval was more concentrated among the poor. Decomposition analysis indicated that wealth quintiles (74.2%), administrative regions (26.4%), and not listening to the radio (5.6%) were the major contributors to the pro-poor socioeconomic inequalities in short birth interval.

**Conclusion:**

There was a pro-poor inequality of short birth interval in Ethiopia. Strengthening the implementation of poverty alleviation programs may improve the population’s socioeconomic status and reduce the associated inequality in short birth interval.

## Introduction

Health inequality is defined as a systematic difference in health across individuals or according to socially relevant groupings such as between more and less advantaged groups [1–3]. Globally, health inequities create one of the main challenges for public health [4]. Socioeconomic inequalities in health and health-related outcomes are particularly common in developing countries [5–7], where the poor are disproportionately affected. As a result, addressing health inequality has become a top priority intervention area for international organizations such as the World Health Organization (WHO) [8, 9], the United Nations Development Programme [10], and the World Bank [11]. For instance, one of the Sustainable Development Goals (SDGs) set by the United Nations (i.e., Goal 10) aims to reduce inequality within and among countries [12, 13]. Monitoring health inequalities help countries track their progress towards the SDG and ensure their disadvantaged or hard-to-reach populations are not left behind [14, 15].

Although reducing inequality is the current central objective of health policy in many countries including Ethiopia, progress has been inadequate [16, 17]. Studies in Ethiopia, for instance, have reported that financially disadvantaged women were less likely to use contraceptives [18]. Similarly, there are high inequalities in access to healthcare resources, in favor of advantaged populations [19]. In contrast, people of low socioeconomic status are less likely to use maternal health services [20, 21].

Previous studies have documented the adverse maternal and child health outcomes associated with short birth interval, defined as inter-birth interval of less than 33 months [22], such as preeclampsia, labor dystocia, low birth weight, preterm birth, congenital anomalies, and infant mortality [23–29]. There is, however, limited knowledge regarding the socioeconomic inequalities related to short birth interval in Ethiopia. Quantifying and characterising socioeconomic inequalities in short birth interval can help policy makers and public health planners target specific groups of women at risk to reduce the burden of short birth interval on maternal and child wellbeing. This study extends on our previous work [30], which identified the individual- and community-level determinants of short birth interval. Our previous study [30] found that women from the poorest, poorer, middle, and richer households were at increased risk of short birth interval compared to women from the richest household. This finding was inconclusive and may not provide precise information to target intervention. Similarly, the small scale studies [31–33] performed in Ethiopia have been inconclusive regarding the association between wealth status and the risk of short birth interval. While one of the studies [33] identified women from the richest households were at higher odds of experiencing short birth interval, the other [31, 32] revealed that women from the poorest, poorer, middle, and richer households were at increased risk of short birth interval.

Unlike the aforementioned previous studies [30–33] performed in Ethiopia, the current study aims to answer the following research questions: “what type of socioeconomic inequality (pro-poor or pro-rich or no inequality problem) in short birth interval is observed in Ethiopia, and what are the factors contributing to any observed socioeconomic inequality?”

The existing empirical studies [30–33] estimated odds ratios as a measure of association between wealth status and short birth interval. Although convenient, concentration index (CI) better allows inequalities to be estimated across the whole population (i.e., in a cumulative share of women ranked by income) using a single metric [34]. Additionally, while the CI can be decomposed to a range of variables that drive the income-related inequality [34, 35], this may not be true for the odds ratio that mainly quantifies the strength of association between wealth index and short birth interval. Moreover, the previous studies [30–33, 36, 37] did not identify factors that derive the socioeconomic inequalities in short birth interval, which mainly can be achieved through decomposing the concentration index.

A simple but comprehensive estimate of the socioeconomic inequality in short birth interval and its contributors is needed to inform policy makers and public health planners for targeted action. In this regard, the CI and decomposition analyses [34, 38] were used in the current study. Concentration index provides precise information regarding whether the socioeconomic inequality in short birth interval is disproportionately concentrated among the poor (pro-poor inequality; negative CI) or the rich (pro-rich inequality; positive CI) or neither of them (no inequality problem; zero concentration index) [11, 35, 39]. Decomposition analysis, on the other hand, helps to identify factors that contributed to the observed socioeconomic inequality [11, 34, 40]. Furthermore, decomposition analysis can provide information about the responsiveness of short birth interval to the change in the determinants variable (i.e., elasticity), which is vital for prioritizing interventions. The use of CIs and decomposition analysis as a means of quantifying the socioeconomic inequalities of health outcomes and its determinants, respectively, are also documented elsewhere [7, 35, 41–46]. The aim of this study is, therefore, to assess the socioeconomic inequality of short birth interval and identify its contributing factors in Ethiopia.

## Methods

### Study design and sampling

The data for this study were extracted from the 2016 Ethiopia Demographic and Health Survey (EDHS). The 2016 EDHS is the fourth and most recent nationally representative Demographic and Health Survey (DHS) in the country. This cross-sectional survey is carried out every 5 years to provide health and health-related indicators for the country as a whole, for urban and rural areas separately, and for each of the nine regions and the two administrative cities. The survey employed a two-stage stratified cluster sampling method. The detailed sampling procedure is presented in the full EDHS report [47]. A total of 8448 ever-married women who had reported at least two live births during the 5 years preceding the survey were included in analyses. When women had more than two births in the 5 years preceding the survey, the birth interval for the two most recent births was considered in this current study.

### Measurement

Socioeconomic inequality in short birth interval was the outcome variable in this study. Birth interval was defined as the time between the birth of the child under study (index child) and the immediately preceding birth [22]. In the current study, birth interval data were collected through extracting the date of birth of women’s biological children from a document such as children’s birth /immunization certificate, and/or asking information regarding their children’s date of birth from the women. Mothers were asked to confirm the accuracy of the information before documenting children’s date of birth from children’s birth/immunization certificates. This crosschecking was performed to avoid errors, since in some cases the documented birth date may represent the date when the birth was recorded, rather than the actual birth date. In the absence of children’s birth certificates, information regarding children’s date of birth was obtained from their mothers. Birth interval was computed in months. Further information regarding birth interval data collection can be found elsewhere [48]. Short birth interval was defined as a birth-to-birth interval of less than 33 months [22].

The socioeconomic inequality of short birth interval can be expressed as the covariance between short birth interval and the fractional rank in the living standards distribution (wealth index in this case). It was then classified into either pro-poor or pro-rich or no inequality problem. Detailed explanation about estimating the socioeconomic inequality of short birth interval can be found in the data analysis section of this paper.

Key explanatory variables included in the decomposition analysis were selected after reviewing relevant literature [30–33, 36, 49, 50]. These were maternal age at first marriage, maternal age at birth of the preceding child, maternal education level, maternal occupational status, wealth index (as a measure of socioeconomic status), place of residence, administrative regions, the total number of children born before the index child, watching television, listening radio, and reading newspapers (see supplementary Table 1).

### Socioeconomic status measure

Wealth index was used as a measure of the socioeconomic status of the household. Households were given scores based on the number and kinds of consumer goods they own, ranging from a television to a bicycle or car, in addition to housing characteristics such as type of drinking water source, flooring materials, toilet and sanitation facilities. A weight or factor score generated through principal components analysis was given to each household asset which was believed to be indicative of the wealth status and for which data was collected. Then standardization of the resulting asset scores to a standard normal distribution with a mean of zero and a standard deviation of one was made. A standardized score for each asset, which varies depending on the household possession status of the asset, was assigned for each household. The scores were summed by the households and the total score of the household was used to rank the individuals. The sample was then divided into population quintiles, that is, five groups, each comprising 20% of the population. The lowest 20% quintile was assigned to the poorest households, the next 20% quintile to the poor households, followed by another 20% quintile for the middle-class households, and finally the top 40% quintile for the rich and richest households. Literature has documented that wealth quintile represents a more long-term (permanent) economic status than either income or consumption does and it is also much easier to implement [51, 52]. Further explanations about the measure of the wealth index have been described in the DHS documents [47, 51–53].

### Data analysis

Descriptive statistics, the frequency with percent, were used to illustrate the distribution of respondents’ background characteristics. *P*-values were computed using Pearson’s chi-squared test. Sampling weight was used throughout the analyses (descriptive, concentration index, and decomposition analyses) to adjust for the non-proportional allocation of the sample to different regions, to their urban and rural areas, and the possible differences in response rates. It ensures the actual representativeness of the survey results at both the national and domain levels. Further explanation of the weighting procedure can be found in the EDHS report [47].

**Fig. 1:**
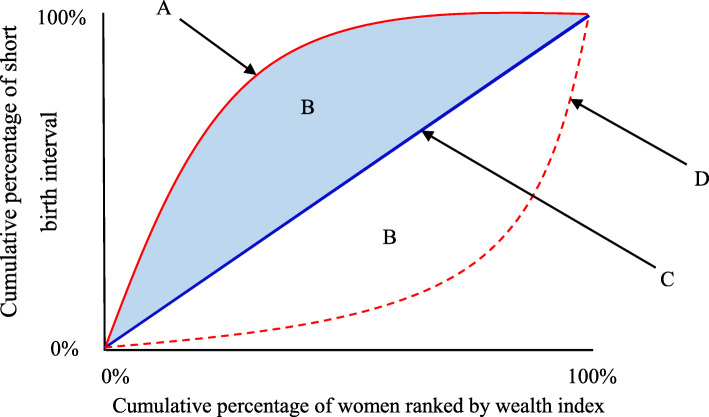
Components of the concentration curve. A = *Concentration curve indicating the pro-poor socioeconomic inequality*; B = *Area between the concentration curve and the line of equality*; C = *Line of equality*; D = *Concentration curve indicating pro-rich socioeconomic inequality*

A concentration index (CI) was computed to measure the socioeconomic inequality in short birth interval. The concentration index is a relative measure of inequality and is defined as twice the area between the concentration curve and the line of equality (the 45-degree line) (i.e., represented by B in Fig. 1) [34, 38]. Mathematically, CI (the covariance between the health variable, short birth interval in this case, and the fractional rank in the living standards distribution (wealth index in this case)) can be written as [11, 54]:

$$ \mathrm{C}=\frac{2}{\upmu} \operatorname {cov}\ \left(\mathrm{h},\mathrm{r}\right) $$

Where c represents concentration index, *μ* is the mean health variable (proportion of short birth interval in this case), *h* represents health variable (short birth interval), and *r* is the cumulative percentage that each woman represents over the total population after ranking short birth interval by the wealth index. For an unbound variable, the index ranges between − 1 and 1 while it ranges from μ − 1 to 1 − μ for bounded variables [55]. Multiplying the value of the concentration index by 75 gives the percentage of the health variable that would need to be (linearly) redistributed from the richer half to the poorer half of the population (in the case that health inequality favors the rich) to arrive at a distribution with an index value of zero (i.e., perfect equality) [39].

To consider the bounded nature of binary health variables, such as short birth interval, Erreygers [56] proposed a modified version of the concentration index (Erreygers normalized concentration index, ECI). Thus, we reported the ECI with the standard error (SE) in this study. The ECI is defined as:

$$ ECI\kern0.5em =\kern0.5em 4\ast \mu \ast CI(y) $$

where *ECI* is Erreygers concentration index, *CI*(*y*) is the generalized concentration index and *μ* is the mean of the health variable (proportion of short birth interval in this case).

Concentration curves were used to graphically depict the socioeconomic related inequality in short birth interval. The curves demonstrate the cumulative percentage of short birth interval on the y-axis against the cumulative percentage of women ranked by the wealth index on the x-axis, sorted from the poorest to the richest. In the case when there is no socioeconomic-related inequality (perfect equity), the concentration index is zero. In other words, if everyone, irrespective of her living standards, has exactly the same value of the short birth interval, the concentration curve lies at a 45-degree line, running from the bottom left-hand corner to the top right-hand corner. This is known as the line of equality (represented by C in Fig. 1). When the concentration index takes a negative value, the curve lies above the line of equality, indicating a disproportionate concentration of the health variable among the poor (pro-poor) (represented by A in Fig. 1). In contrast, the curve lies below the line of equality when the concentration index takes a positive value indicating the outcome variable is concentrated among the rich (pro-rich) (represented by D in Fig. 1) [11, 34]. The further the concentration curve (i.e., A or D in Fig. 1) lies from the diagonal line, the greater the degree of inequality in short birth interval across quintiles of economic status [34, 38].

While visual inspection of a concentration curve in comparison with the line of equality may give an impression of whether there is dominance (i.e., whether the concentration curve lies above or below the line of equality), this inspection is not sufficient to conclude whether or not the concentration curve dominance is statistically significant. Therefore, a dominance test was performed to examine the statistical significance of the difference between the concentration curve and the line of equality (45-degree or diagonal line). It helps to identify significance of difference between ordinates of curves at a number of quantiles. To achieve this, a multiple comparison approach that considered multiple testing using critical values from the Studentized Maximum Modulus (SMM) distribution was performed. If a critical value for 5% significance level to test significance between co-ordinates is used, then as the number of points of comparison is increased, the more likely it becomes that the null hypothesis could be falsely rejected. In contrast, if too few points of comparison were considered, dominance across the full range of distribution cannot be tested. Hence, it is necessary to correct the critical value for the number of comparisons made. The SSM distribution takes account of the number of points of comparison. Thus, 19 equally spaced quantile points, a recommended points choice, and *p*-values of less than 0.05 for declaring statistical significance were used [11]. Further information about the dominance test can be found elsewhere [11].

Decomposition of the concentration index was also performed to identify the relative contribution of various factors to the socioeconomic-related inequality in short birth interval [11, 34, 40]. For any linear additive regression model of health outcome (y) [38], such as:

$$ y=\alpha +{\sum}_k{\beta}_k{x}_k+\varepsilon $$

the concentration index for y, C, is given as:

$$ C=\kern0.5em \sum \limits_k\left(\frac{\upbeta_{\mathrm{k}}{\overline{x}}_k}{\mu}\right){C}_k+\frac{G{C}_{\varepsilon }}{\mu } $$

Where *y* is a health outcome variable (socioeconomic inequality of short birth interval in this case), *x*_*k*_ is a set of socioeconomic determinants of health outcome, *α* is an intercept, *β*_*k*_ is the coefficient of *x*_*k*_, *μ* is the mean of *y*, ${\overline{x}}_k$ is the mean of *x*_*k*_, *C*_*k*_ is the concentration index for *x*_*k*_ (defined analogously to C), and *GC*_*ε*_ denotes the generalized concentration index for the error term/residual term (*ε*), $\left(\frac{\beta_k{\overline{x}}_k}{\mu}\right)$ represent the elasticity (*η*_*k*_) of *y* (short birth interval) with respect to ${\overline{x}}_k$, which is the impact of each determinant on the probability of short birth interval [11, 40]. The residual (*ε*) reflects the inequality in short birth interval that cannot be explained by systematic variations across income groups in the *x*_*k*_, which should approach zero for a well specified model [11, 40]. Sampling weight was considered while performing the decomposition analysis. In this current study, the health variable, short birth interval was measured as a binary variable taking the value of one or zero, depending on the experience of short birth interval and non-short birth interval, respectively. The linear probability model (LPM) and non-linear logit model are the two standard models for a binary variable. The estimate from the LPM are easily interpretable and provide the probability of short birth interval that are < 0 or > 1 but has heteroscedastic errors. The choice of the model, the LPM or the non-linear logit, was made by performing the linktest [11, 57, 58]. The squared linear prediction with no explanatory power shows a correctly specified model. Accordingly, the logit specifications and a survey-specific logit model was chosen.

Stata version 14 *(StataCorp. Stata Statistical Software: Release 14. College Station, TX: StataCorp LP. 2015)* was used for the statistical analysis.

## Results

### Background characteristics of the study participants

A total of 8448 women were included in the analysis. The prevalence of short birth interval in Ethiopia was 45.8% (95% CI: 42.91–48.62).

Table 1 illustrates the weighted proportion of short birth interval based on the background characteristics of the study participants. A large proportion of women with short birth interval were aged 19 years or under at their first marriage (79.4%), uneducated (76.4%), unemployed (75.1%), rural residents (94.0%), from the poorest households (28.8%), and had five and more children born before the index child (43.1%).

**Table 1:** Weighted^*^ proportion of short birth interval by selected background characteristics of respondents, EDHS 2016

Variables	Weighted proportion		*P* value
	Non-short birth intervaln(%)	Short birth intervaln(%)	
Maternal age at first marriage (years)			
≤ 19	3499 (83.6)	3324 (79.4)	< 0.001
20–24	600 (12.7)	697 (16.3)	
25–29	116 (2.8)	142 (3.6)	
30+	46 (0.9)	24 (0.7)	
Maternal age at birth of the preceding child (years)			
≤ 19	712 (16.7)	598 (13.8)	< 0.001
20–24	1383 (31.3)	1391 (32.6)	
25–29	1117 (26.6)	1143 (27.9)	
30–34	723 (16.9)	706 (16.4)	
35+	326 (8.5)	349 (9.3)	
Maternal educational level			
No education	2871 (71.9)	3201 (76.4)	< 0.001
Primary	1019 (22.9)	779 (20.7)	
Secondary	250 (3.4)	131 (1.8)	
Higher	121 (1.7)	76 (1.1)	
Maternal occupation			
Not working	2949 (71.1)	3164 (75.1)	< 0.001
Working	1312 (28.9)	1023 (24.9)	
Wealth quintiles			
Poorest	1348 (22.0)	1999 (28.8)	< 0.00
Poorer	759 (22.1)	719 (24.8)	
Middle	658 (21.0)	545 (21.5)	
Richer	589 (19.1)	466 (16.6)	
Richest	907 (15.8)	458 (8.3)	
Place of residence			
Urban	818 (11.3)	468 (6.0)	< 0.001
Rural	3443 (88.7)	3719 (94.0)	
Regions			
Tigray	525 (7.5)	263 (4.3)	< 0.001
Afar	294 (0.7)	534 (1.4)	
Amhara	578 (25.2)	222(11.1)	
Oromia	641(39.6)	666 (50.9)	
Somali	343 (2.4)	940 (8.0)	
Benishangul-Gumuz	363 (1.0)	372 (1.2)	
**SNNPR	566 (20.7)	478 (21.5)	
Gambella	338 (0.3)	219 (0.2)	
Harari	231(0.2)	225 (0.2)	
Addis Ababa	188 (2.1)	64 (0.8)	
Dire Dawa	194 (0.3)	204 (0.4)	
Total number of children born before the index child			
≤ 2	1538 (34.3)	1146 (26.8)	< 0.001
3–4	1333 (30.7)	1305 (30.1)	
≥ 5	1390 (35.0)	1736 (43.1)	
Watched television			
Yes	946 (18.9)	538 (12.9)	< 0.001
No	3315 (81.1)	3649 (87.1)	
Listened to radio			
Yes	1086 (26.6)	734 (23.0)	< 0.001
No	3179 (73.4)	3453 (77.0)	
Read newspapers			
Yes	296 (5.6)	143 (3.8)	< 0.001
No	3965 (94.4)	4044 (96.2)	

*Weighted proportion was computed after applying sample weights; Pearson’s chi-squared test was used to compute the *p*-values

**SNNPR Southern Nations, Nationalities, and Peoples’ Region, *EDHS* Ethiopia Demographic and Health Survey

### Socioeconomic inequality of short birth interval

**Fig. 2:**
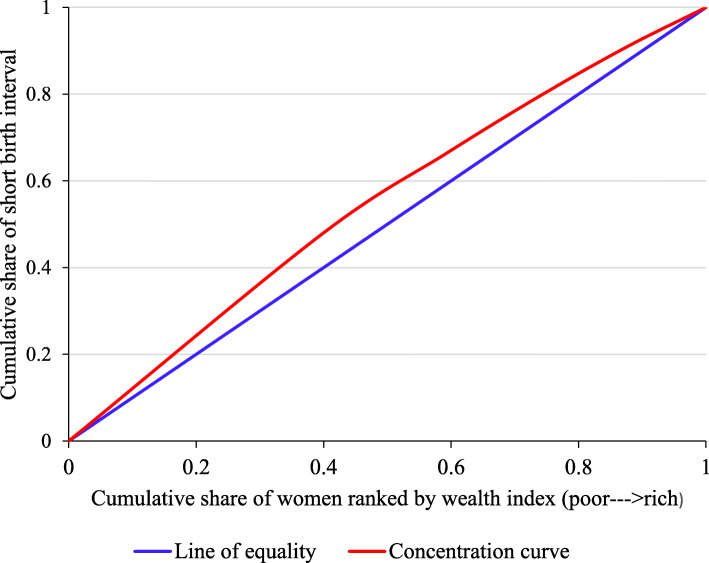
Concentration curve for short birth interval in Ethiopia, EDHS 2016. EDHS = Ethiopia Demographic and Health Survey

The weighted Erreygers normalized concentration index (ECI) for short birth interval was − 0.0478 (SE = 0.0062) and differed significantly from zero (*P* < 0.0001). This indicates that short birth interval was disproportionately concentrated among the poor. Figure 2 depicts the concentration curve of short birth interval in Ethiopia. The concentration curve illustrates the relationship between the cumulative proportion of women ranked by the household wealth index on the horizontal axis and the cumulative proportion of short birth interval on the vertical axis. The 45-degree diagonal line represents the line of perfect equality, which is equivalent to a concentration index equal to zero. The current study found that the concentration curve laying above the line of perfect equality indicating a pro-poor inequality meaning short birth interval was disproportionately concentrated amongst women from poorer households. The dominance test also showed the concentration curve for short birth interval dominated the line of equality (*p*-value < 0.001).

### Decomposing the socioeconomic inequality in short birth interval

While computing the concentration index to identify the extent of socioeconomic-related inequality in short birth interval is useful, wealth quintiles are only one of the determinants that influence socioeconomic inequality, either directly or indirectly. To identify how much of the measured socioeconomic-related inequality in short birth interval was due to wealth quintiles and due to other variables, decomposition analysis was performed [11, 34, 40].

Table 2 presents findings from the decomposition analysis. The decomposition analysis shows the contributions of individual determinant to the overall socioeconomic inequality of short birth interval. The column ‘Elasticity’, a unit-free measure of partial association, denotes the change in the dependent variable (socioeconomic inequality in short birth interval in this case) associated with a one-unit change in the explanatory variables [45, 58]. Specifically, it represents the responsiveness of the health outcome (socioeconomic inequality in short birth interval in this case) to a change in the determinant variable [40]. A positive and negative sign in elasticity indicates an increasing or decreasing change of short birth interval in association with a positive change in the determinant. For instance, the value of elasticity for urban resident women was − 0.0273, indicating that a 1% change in women’s place of residence from a rural area to the urban area will result in a − 2.7% change (i.e., reduction) in socioeconomic inequality of short birth interval. While a 1% change in the region of residence from Dire Dawa to Somali (1.6%) increase the pro-poor socioeconomic inequality of short birth interval, the change in a region of residence from Dire Dawa to Tigray (− 2.6%), Amhara (− 10.6%), Benishangul-Gumuz (− 0.1%), SNNPR (− 4.1%), Gambella (− 0.1%), and Addis Ababa (− 0.3%) reduced the pro-poor socioeconomic inequality in short birth interval.

**Table 2:** Contributing factors of inequality in short birth interval in Ethiopia, EDHS 2016

Variables	Coefficient	Elasticity	Concentration index	Standard error^ϯ^	Absolute contribution	Percentage contribution (%)
**Maternal age at first marriage** (ref: 30+ years)						
≤ 19	0.0873	0.1560	− 0.0021	0.0649	− 0.0003	
20–24	0.1581^**^	0.0495	0.0106	0.0678	0.0005	
25–29	0.2047^*^	0.0141	− 0.0261	0.0737	− 0.0004	
Subtotal					**−0.0002**	**0.4**
**Maternal age at birth of the preceding child** (ref: 35+ years)						
≤ 19	0.0283	0.0095	− 0.0439	0.0316	− 0.0001	
20–24	0.0422	0.0295	− 0.0086	0.0224	− 0.0002	
25–29	0.0130	0.0077	0.0122	0.0203	0.0001	
30–34	−0.0175	− 0.0064	0.0227	0.0224	−0.0001	
Subtotal					**−0.0003**	**0.6**
**Maternal educational level** (ref: No education)						
Primary	0.0149	0.0072	0.1359	0.0154	0.0006	
Secondary	0.0394	0.0023	0.3326	0.0260	0.0008	
Higher	0.1580^*^	0.0049	0.3621	0.0412	0.0018	
Subtotal					**0.0032**	**−6.7**
**Maternal occupation** (ref: Working)						
Not working	0.0333^*^	0.0531	−0.0226	0.0119	−0.0012	
Subtotal					**−0.0012**	**2.5**
**Wealth quintiles** (ref: Richest)						
Poorest	0.1201^*^	0.0622	−0.7369	0.0183	−0.0258	
Poorer	0.0920^*^	0.0457	−0.3107	0.0198	−0.0202	
Middle	0.0799^*^	0.0342	0.1108	0.0193	0.0038	
Richer	0.0477^**^	0.0190	0.4982	0.0222	0.0067	
Subtotal					**−0.0355**	**74.2**
**Place of residence** (ref: Rural)						
Urban	−0.1402^*^	−0.0273	− 0.3215	0.0180	0.0089	
Subtotal					**0.0089**	**−18.6**
**Regions** (ref: Dire Dawa)						
Tigray	−0.2007^*^	− 0.0264	−0.1049	0.0314	0.0028	
Afar	0.0543	0.0012	−0.8091	0.0313	−0.0009	
Amhara	−0.2596^**^	− 0.1065	−0.0091	0.0313	0.0009	
Oromia	−0.0361	−0.0354	0.0679	0.0239	−0.0024	
Somali	0.1496^*^	0.0161	−0.6359	0.0272	− 0.0102	
Benishangul-Gumuz	−0.0539	−0.0013	− 0.1488	0.0299	0.0002	
SNNPR^a^	−0.0900^*^	−0.0415	0.0669	0.0261	−0.0018	
Gambella	−0.1669^*^	−0.0008	− 0.4752	0.0313	0.0004	
Harari	−0.0099	−0.0001	0.0685	0.0295	−0.0001	
Addis Ababa	−0.1029^*^	−0.0034	0.4347	0.0367	−0.0015	
Subtotal					**−0.0126**	**26.4**
**Total number of children born before the index child** (reference: 5+)						
≤ 2	−0.0965^*^	− 0.0651	0.0289	0.0192	−0.0019	
3–4	−0.0606^*^	−0.0402	− 0.0315	0.0146	0.0013	
Subtotal					**−0.0006**	**1.3**
**Watched television** (ref: Yes)						
No	0.0308	0.0566	−0.0322	0.0188	−0.0018	
Subtotal					**−0.0018**	**3.8**
**Listened to the radio** (ref: Yes)						
No	0.0204^**^	0.0336	−0.0820	0.0140	−0.0027	
Subtotal					**−0.0027**	**5.6**
**Read newspapers** (ref: Yes)						
No	0.0376	0.0785	−0.0147	0.0230	−0.0011	
Subtotal					**−0.0011**	**2.3**
**Explained**					**−0.0435**	**91.8**
**Residual**					**0.0039**	**8.2**

ref = reference group; ^a^*SNNPR* Southern Nations, Nationalities, and Peoples’ Region, *EDHS* Ethiopia Demographic and Health Survey

Note: Estimates are weighted; ^ϯ^ bootstrapped standard errors with 1000 replications; * and ** indicate significance at less than 0.01 and 0.05 levels, respectively

The column ‘Concentration index’ represents the distribution of the determinants itself with reference to wealth quintiles. The positive or negative sign of the CI indicates that the determinants of inequality were concentrated among the rich or poor households, respectively. The CIs in this study, for instance, found that women aged between 25 and 29 years at their first marriage, unemployed women, and urban resident were more likely to be concentrated in the lower tail of the wealth distribution. In contrast, women aged between 20 and 24 at their first marriage and women who had attended higher education were more likely to be concentrated in the upper tail of the wealth distribution.

**Fig. 3:**
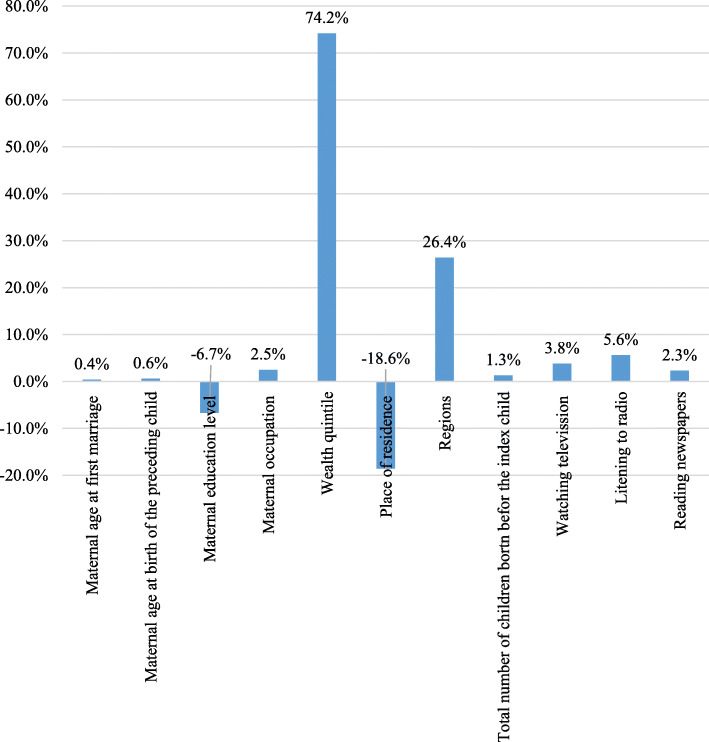
The percentage contribution of selected determinants to the overall socioeconomic inequality in short birth interval in Ethiopia, EDHS 2016. EDHS = Ethiopia Demographic and Health Survey

The percentage contribution represents the relative contribution of each determinant included in the model to the overall socioeconomic-related inequality in short birth interval. A positive percentage contribution shows a specific factor that results in increasing the observed socioeconomic inequality. In contrast, a negative percentage contribution indicates the one that results in decreasing the observed socioeconomic inequality. Our study found that the socioeconomic inequality in short birth interval was largely driven by the wealth itself (74.2%), as shown by the adjusted percentage contribution of inequality. The regions where women lived, in general, were responsible for 26.4% of the socioeconomic inequality. Not listening to radio also contributed to the socioeconomic inequalities in short birth interval, explaining 5.6% of the overall inequality. Factors such as maternal age at first marriage and maternal age at birth of the preceding child explained only a small percentage of the inequalities. Place of residence was the primary contributor to the reduction (-18.6%) of socioeconomic inequality in short birth interval followed by maternal education (-6.7%). Figure 3 also summarizes the percentage contributions of each determinant of the socioeconomic status related inequalities in short birth interval. The figure generally demonstrated that the wealth quintile had a greater contribution (74.2%) to the overall socioeconomic inequality in short birth interval.

The value for the error term/residual component of the model was 0.0039. Since the residual approach to zero, the decomposition analysis provided a well-specified model where the socioeconomic-related inequality in short birth interval that was not explained by systematic variation in the determinants across wealth groups was too small [11].

## Discussion

To our knowledge, this is the first study to quantify the socioeconomic inequality in short birth interval and its drivers in Ethiopia. The findings showed that short birth interval was more concentrated among the poor. Wealth quintiles, administrative regions, and not listening to the radio were the major contributors to the pro-poor socioeconomic inequalities in short birth interval. The findings of the current study can help policy makers and program planners address the inequality issue to achieve the SDG 10 target. Generally, this study is a valuable addition to the scientific literature on inequality and fills the knowledge gap on the socioeconomic disparities in short birth interval, which is one of the contemporary maternal health issues.

In this current study, it was found that short birth interval is in favor of women from poor households. However, this does not imply that short birth interval does not occur among the rich. Rather it revealed short birth interval is disproportionately concentrated among the poor. Evidence has also shown that health and poverty are intimately intertwined, and poor health is more prevalent among the poor community than the better-off group [4, 59]. The finding could imply that socioeconomically disadvantaged women may not be able to access health information, could have less knowledge about family planning, and could be less autonomous to decide the number and timing of their children. This, in turn, may deteriorate women’s ability to enjoy a healthy life such as optimum birth interval. Failure to avoid this inequality may result in social injustices in addition to its adverse health consequences to the women and their children. Therefore, to help women control their childbirths spacing, beyond providing family planning service free of charge, there is a need to intensify programmes that improve the socioeconomic status of disadvantaged women.

It was found that wealth quintiles itself (74.2%) were the predominant determinant of the overall socioeconomic inequality in short birth interval. Evidence from a cross-country study performed in 18 Sub-Saharan countries has also revealed that wealth was the single most important driver of inequality in maternal healthcare utilization [60]. Prior studies [20, 41, 61, 62] on related maternal health issues in Ethiopia and other developing countries also documented similar findings. Wealth status could affect birth interval of women through its effect on accessing family planning information and services. Literature has documented that the rate of modern contraceptive use, a mechanism used to control birth interval, was higher among economically and educationally advantaged women [63, 64]. In addition, differences in access to family planning, information, and direct contact with field workers are associated with the wealth gradient [65]. Moreover, income disparity may create differences in other health determinants, such as people’s ability to pay for the indirect cost for transportation fees to the health facility and other opportunistic costs.

The current study also demonstrated that administrative regions of residence were the second most important contributors (26.4%) to socioeconomic inequality in short birth interval. Literature has also shown that poverty is much more widespread and severe among pastoralists and the agro-pastorals region of Ethiopia due to their scattered and nomadic lifestyle [66, 67], which create a challenge to provide basic public services. The disparities in inequality among administrative regions could be due to the wide variations in sociodemographic and cultural characteristics, distribution of health facilities, and developmental activities among regions.

We found that place of residence contributed to the reduction (− 18.6%) of socioeconomic inequality in short birth interval. In spite of the government efforts to deploy a large number of community health extension workers and expand health facilities including health posts in most of the rural areas [67–69], the inequality in health outcomes such as short birth interval based on place of residence continue. The previous study has also documented the presence of inequality in rural than in urban areas [67]. Furthermore, the existing evidence also suggests that the place of residence has a significant impact on maternal health care utilization [62, 70, 71] adversely affecting rural women. Women who resided in an urban area are likely to access better job opportunities, health information, health facilities, and family planning services than their counterparts.

It was found that not listening to radio also contributed to the inequalities in short birth interval, explaining 5.6%. The 2016 EDHS has reported that radio was the most frequently accessed type of media [47]. Other studies [72–74] have revealed that women who are exposed to radio messages about family planning were more likely to discuss family planning with their spouses and use the services. This may help women and their partners limit and space their childbirths. Women who had no exposure to the radio may miss out on possible resources to improve their health literacy, economic, and social capital.

As a strength, the study used data from a nationally representative and large sample of a population-based survey. The application of a more rigorous decomposition analysis to determine factors contributing to socioeconomic inequality in short birth interval is another strength of the current study.

We acknowledge the following limitations of this study. First, the cross-sectional nature of the study design does not allow us to draw causal inferences. Second, the use of wealth quintile as a measure of socioeconomic status should be considered cautiously. If the standardized living status measurement were used, some of the upper tail of the wealth distribution such as middle and richer wealth quintiles would fail below the poverty line.

## Conclusion and recommendations

There was a pro-poor inequality of short birth interval in Ethiopia. Wealth quintiles, regions of residence, and not listening to the radio were the major contributors to the pro-poor socioeconomic inequalities in short birth interval.

Households’ wealth status, administrative regions, and women’s media exposure particularly to the radio should be interventions priority targeting the reduction of socioeconomic-related inequality in short birth interval. Accelerating the implementation of poverty alleviation projects, such as implementing social safety net programs [75] and creating job opportunities could be among the key mitigation to pull out the disadvantaged women from poverty, and address the socioeconomic inequality. Recent evidence has shown that social safety nets help people escape extreme poverty, close the poverty gap, and reduce inequality [75]. Equitable economic growth and fair distribution of resources among urban-rural areas and administrative regions of Ethiopia can also help narrow the observed inequality gap. To achieve this, developing administrative regions specific policy should be given much attention. Awareness creation on optimum birth interval and family planning, particularly for women from the lower socioeconomic status, is vital. Improving women’s access and exposure to radio is also recommended.

## Supplementary information

**Additional file 1: Supplementary Table 1.** Key explanatory variables included in the decomposition analysis.

## Data Availability

The dataset is available from The DHS Program repository at the following hyperlink: https://www.dhsprogram.com/data/dataset/Ethiopia_Standard-DHS_2016.cfm?flag=0.

## Abbreviations

CI: Concentration Index
DHS: Demographic and Health Survey
ECI: Erreygers Normalized Concentration Index
EDHS: Ethiopia Demographic and Health Survey
SDGs: Sustainable Development Goals
SE: Standard Error
SMM: Studentized Maximum Modulus
SNNPR: Southern Nations, Nationalities, and Peoples’ Region
WHO: World Health Organization
