# Supplementary material for: Socioeconomic inequality in short birth interval in Ethiopia: a decomposition analysis
Source: BMC Public Health. 2020 Oct 6;20:1504. doi: 10.1186/s12889-020-09537-0 (PMC7542382; doi:10.1186/s12889-020-09537-0)
Supplement: Supplementary file 1 — Additional file 1: Supplementary Table 1. Key explanatory variables included in the decomposition analysis. [file 12889_2020_9537_MOESM1_ESM.docx]

**Supplementary Table 1.** Key explanatory variables included in the decomposition analysis

| **Variable** | **Category/Values** |
| --- | --- |
| Maternal age at first marriage | Maternal age at first marriage categorized as 1=<19; 2=20-24; 3=25-29; 4=30+ |
| Maternal age at birth of the preceding child | Maternal age at birth of the preceding child categorized as 1=<19; 2=20-24; 3=25-29; 4=30-34; 5=35+ |
| Maternal education level | Maximum educational level of women categorized as 1=No Education; 2= Primary; 3=Secondary; 4=Higher |
| Maternal occupation | Current maternal occupational status categorized as 1=Not working; 2=Working |
| Wealth quintile | The wealth index provided with the dataset was used (47). DHS program provides a composite index of household amenities based on the principal component analysis (PCA) and classified the population into quintiles: (1st quintile (Poorest); 2nd quintile; 3rd quintile; 4th quintile and 5th quintile (Richest). |
| Place of residence | Place of residence was categorized in to 1=Urban; 2=Rural |
| Regions | 1=Tigray; 2=Afar; 3=Amhara; 4=Oromia; 5=Somali; 5= Benishangul-Gumuz; 7=SNNPR**; 8=Gambella; 9=Harari; 10=Addis Ababa and 11=Dire Dawa |
| Total number of children born before the index child | Total number of children born before the index child categorized as 1= ≤2, 2= 3 to 4 and 3= ≥5 children |
| Watched television (TV) | Categorized as 1=Yes; 2=No |
| Listen to radio | Categorized as 1=Yes; 2=No |
| Read newspaper | Categorized as 1=Yes; 2=No |

SNNPR**= Southern Nations, Nationalities, and Peoples' Region; EDHS= Ethiopia Demographic and Health Survey
